# Transmission Dynamics of Severe Acute Respiratory Syndrome Coronavirus 2 in High-Density Settings, Minnesota, USA, March–June 2020

**DOI:** 10.3201/eid2708.204838

**Published:** 2021-08

**Authors:** Nicholas B. Lehnertz, Xiong Wang, Jacob Garfin, Joanne Taylor, Jennifer Zipprich, Brittany VonBank, Karen Martin, Dana Eikmeier, Carlota Medus, Brooke Wiedinmyer, Carmen Bernu, Matthew Plumb, Kelly Pung, Margaret A. Honein, Rosalind Carter, Duncan MacCannell, Kirk E. Smith, Kathryn Como-Sabetti, Kris Ehresmann, Richard Danila, Ruth Lynfield

**Affiliations:** Minnesota Department of Health, St. Paul, Minnesota, USA (N. Lehnertz, X. Wang, J. Garfin, J. Zipprich, B. VonBank, K. Martin, D. Eikmeier, C. Medus, B. Wiedinmyer, C. Bernu, M. Plumb, K. Pung, K.E. Smith, K. Como-Sabetti, K. Ehresmann, R. Danila, R. Lynfield);; Centers for Disease Control and Prevention, Atlanta, Georgia, USA (J. Taylor, M.A. Honein, R. Carter, D. MacCannell)

**Keywords:** 2019 novel coronavirus disease, coronavirus disease, COVID-19, severe acute respiratory syndrome coronavirus 2, SARS-CoV-2, viruses, respiratory infections, zoonoses, whole-genome sequencing, transmission, epidemiology, Minnesota, United States

## Abstract

Coronavirus disease has disproportionately affected persons in congregate settings and high-density workplaces. To determine more about the transmission patterns of severe acute respiratory syndrome coronavirus 2 (SARS-CoV-2) in these settings, we performed whole-genome sequencing and phylogenetic analysis on 319 (14.4%) samples from 2,222 SARS-CoV-2–positive persons associated with 8 outbreaks in Minnesota, USA, during March–June 2020. Sequencing indicated that virus spread in 3 long-term care facilities and 2 correctional facilities was associated with a single genetic sequence and that in a fourth long-term care facility, outbreak cases were associated with 2 distinct sequences. In contrast, cases associated with outbreaks in 2 meat-processing plants were associated with multiple SARS-CoV-2 sequences. These results suggest that a single introduction of SARS-CoV-2 into a facility can result in a widespread outbreak. Early identification and cohorting (segregating) of virus-positive persons in these settings, along with continued vigilance with infection prevention and control measures, is imperative.

In the United States, coronavirus disease (COVID-19) has disproportionately affected adults residing in long-term care facilities (LTCFs) (*1*–*5*). Outbreaks in LTCFs have caused high numbers of hospitalizations and deaths. Similar findings have been reported in correctional facilities, where severe acute respiratory syndrome coronavirus 2 (SARS-CoV-2) infection incidence among inmates and staff is ≈5 times greater and age-adjusted mortality rate 3 times greater than that of the general population (*6*–*8*). Workers in high-density workplaces (e.g., meat-processing plants) have similarly been heavily affected; minority populations have been disproportionately affected (*9*–*11*).

The first COVID-19 case in Minnesota was detected on March 6, 2020. Shortly thereafter, COVID-19 outbreaks occurred across the state, including in LTCFs (March 12, 2020) and meat-processing plants (March 15, 2020), followed shortly thereafter by correctional facilities (March 25, 2020). During March 6–June 30, 2020, the Minnesota Department of Health (MDH) identified and responded to 1,060 distinct outbreaks of COVID-19 in LTCFs, comprising 4,421 cases in residents and 3,002 in staff members. In addition, 4 discrete outbreaks in correctional facilities resulted in 382 cases, and 68 outbreaks in meat-processing plants resulted in ≈2,616 cases among employees (data only from persons interviewed and where workplace information was provided); outbreaks in these 3 settings accounted for 31.3% of all identified persons in Minnesota.

For outbreaks in congregate settings and high-density workplaces, confirming the temporal and relational aspects of SARS-CoV-2 transmission was difficult, and the role of intrafacility spread versus multiple introductions was difficult to disentangle on the basis of epidemiologic information alone. Whole-genome sequencing (WGS) of specimens from outbreak case-patients can be used to determine transmission dynamics and relatedness of viral pathogens in infectious disease outbreaks (*12*–*15*). Unprecedented efforts to sequence SARS-CoV-2 genomes have occurred at the local, regional, national, and international levels to investigate potential reinfections (*16*–*19*), nosocomial transmission (*20*), patterns of community spread (G.K. Moreno et al., unpub. data, ) (*21**,**22*), and sources of SARS-CoV-2 introduction without known epidemiologic links (*23*). 

In Minnesota, as part of the Centers for Disease Control and Prevention (CDC) SARS-CoV-2 Sequencing for Public Health Emergency Response, Epidemiology and Surveillance (SPHERES) consortium, the Minnesota Molecular Surveillance of SARS-CoV-2 initiative solicited specimens from outbreak case-patients for sequencing and genetic variation analysis to determine virus transmission patterns in congregate settings and meat-processing plants. To supplement epidemiologic information, assess whether single or multiple introductions were likely to have occurred during a facility outbreak, and evaluate molecular relatedness, we performed WGS on a convenience sample of SARS-CoV-2–positive specimens associated with outbreaks.

## Methods

We chose 3 types of outbreak settings for WGS (LTCFs, correctional facilities, and meat-processing plants) and selected specific facilities partly according to outbreak effect and severity, the need for further clarity regarding transmission patterns, and availability of samples. Selected outbreaks occurred during March 6–June 30, 2020, at 4 unique LTCFs (A–D), 2 correctional facilities (A and B), and 2 meat-processing plants (A and B); cases were identified in persons residing in the same county as meat-processing plant A (community samples A).

At LTCFs, an outbreak was defined as >1 confirmed COVID-19 case in a resident or staff member. At correctional facilities, an outbreak was defined as 1 of the following:

• >2 cases in the inmate population >7 days after intake to a new facility with an epidemiologic link (defined as residing in the same unit or ward within a 14-day period).

• >2 cases in correctional staff members with an epidemiologic link (defined as having the potential to have been within 6 feet for >15 minutes while working in the facility during the 14 days before symptom onset (e.g., worked on the same unit during the same shift). An epidemiologic link also requires that cases among correctional staff neither shared a household nor were identified as close contacts with each other outside the facility during the standard case investigation.

• >1 facility-acquired COVID-19 cases in an inmate (defined as a confirmed diagnosis >14 days after entry to the facility, without exposure during the previous 14 days to another setting where an outbreak was known or suspected).

At meat-processing facilities, an outbreak was defined as >3 laboratory-confirmed COVID-19 cases among facility workers who resided in separate households. On June 1, we added to the definition of an outbreak in meat-processing plants that case onset dates occurred within 14 days of each other.

We defined case-patients at all outbreak locations as persons with a positive SARS-CoV-2 result according to reverse transcription PCR (RT-PCR), determined by using the original CDC protocol (*24*). We collected epidemiologic data (sex, age, symptom status, symptom onset date, residence, occupation, and potential source of exposure) by interviewing persons with laboratory-confirmed SARS-CoV-2.

The MDH Public Health Laboratory (PHL) performed WGS on available specimens positive for SARS-CoV-2 by RT-PCR, collected March 6–June 30, 2020. Specimens were obtained from the nasopharynx, anterior nares, or oropharynx. SARS-CoV-2 RNA extracts were acquired either as residuals from clinical testing at the MDH PHL or from other clinical laboratories serving Minnesota residents. We created cDNA and tiled amplicons as described in the ARTIC Network nCoV-2019 sequencing protocol (*25*). We prepared Illumina sequencing libraries for next-generation sequencing according to the Nextera DNA Flex protocol created by the State Public Health Bioinformatics Group (StaPH-B) (*26*) and performed sequencing by using 2×250 bp Illumina V2 chemistry on MiSeq instruments (https://www.illumina.com). Consensus SARS-CoV-2 genome sequences for each specimen were generated with the StaPH-B Toolkit Monroe pipeline (https://staph-b.github.io/staphb_toolkit/workflow_docs/monroe). We individually reviewed assembled SARS-CoV-2 genomes in Geneious Prime 2019.2.1 (https://www.geneious.com) and discarded genomes with gaps >125 nt.

We used the Augur toolkit (*27*) to align SARS-CoV-2 genome consensus sequences, generate phylogenetic trees, and incorporate epidemiologic sequence metadata. We aligned genomes with MAFFT version 7.310 with options “–keeplength–reorder–anysymbol–nomemesave–adjustdirection” (*28*). Variation in sequences identified in the first 54 and last 67 bases of the Wuhan-Hu-1 reference sequence (GenBank accession no. MN908947.3) was masked during tree generation because of the inability of the tiled-amplicon sequencing approach to reliably generate sequence in those regions. We used IQ-TREE version 1.6.1 to create phylogenetic trees with parameters “-ninit 2 -n 2 -me 0.05” (*29*). Output from Augur was visualized by using Auspice as hosted by the nextstrain team (http://auspice.us) (*27*). The resulting trees were visualized with the Interactive Tree of Life (*30*); branch lengths rounded and scaled represent mutations from the reference. Pangolin lineages for all samples were retrieved after assemblies were submitted to GISAID (https://github.com/cov-lineages/pangolin) (*27**,**31*).

We defined genetically closely related sequences (i.e., clusters) as cases that were both associated epidemiologically with a known outbreak and that formed a monophyletic clade on the statewide phylogenetic tree. Branch lengths were scaled to represent the number of single-nucleotide mutations.

In accordance with federal human subjects protection regulations at 45 CFR §46.101c and §46.102d and with the Guidelines for Defining Public Health Research and Public Health Non-Research, a human subjects protection coordinator at CDC and the MDH reviewed the project. They determined it to be a nonresearch, public health response exempt from institutional review board evaluation.

## Results

**Figure 1:**
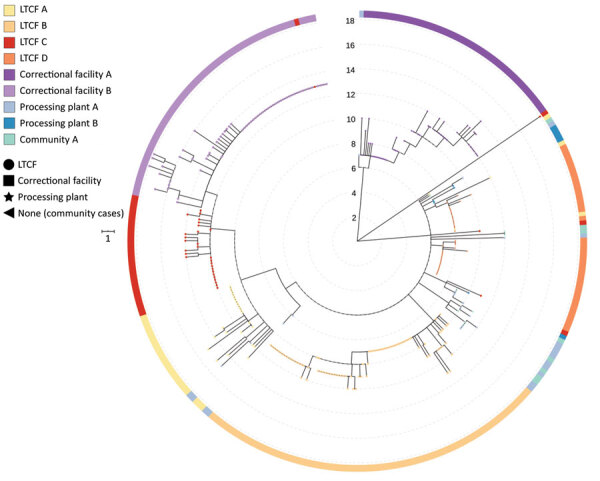
Phylogenetic tree of severe acute respiratory syndrome coronavirus 2 associated with selected outbreaks in Minnesota, USA, March 6–June 30, 2020. IQ-TREE (*29*) was used with the general time reversible substitution model for tree generation. Branch lengths were scaled to represent number of single-nucleotide mutations as shown in the scale key. LTCF, long-term care facility.

As of June 30, 2020, we had successfully conducted WGS and phylogenetic analysis of 468 total samples, 319 (68.2%) of which were associated with the 8 outbreaks, constituting 14.4% of the 2,222 total positive cases identified from outbreaks in Minnesota through June 2020. Specimens were obtained from staff and residents from 4 LTCFs (180 [35.6%] specimens from 505 case-patients were sequenced), staff and inmates from 2 correctional facilities (110 [20.2%] specimens from 544 case-patients were sequenced), and employees at 2 meat-processing plants, along with community case-patients (29 [2.5%] samples from 1,173 identified case-patients) (Table). Among most sequenced specimens, virus spread was associated with a single genetic sequence unique to each outbreak facility at 3 LTCFs and both correctional facilities. At a fourth LTCF, outbreak cases were associated with 2 distinct sequences. In contrast, cases associated with outbreaks in the 2 meat-processing plants were represented by multiple SARS-CoV-2 sequences. (Figure 1)

**Table:** Features of outbreaks and convenience samples of specimens collected and characterized by whole-genome sequencing at LTCFs, correctional facilities, and meat-processing plants in Minnesota, USA, March 6–June 30, 2020*

Outbreak facility	Total confirmed outbreak cases at facility, no.	Total samples successfully sequenced from facility, no. (%)	Role at facility	Total outbreak cases at facility confirmed by role, no.	Total samples successfully sequenced by role, no. (%)
LTCF					
A	89	27 (30.3)	Staff	38	10 (26.3)
			Residents	51	17 (33.3)
B	190	82 (43.2)	Staff	76	5 (6.6)
			Residents	114	77 (67.5)
C	139	32 (23.0)	Staff	56	23 (41.0)
			Residents	83	9 (10.8)
D	74	39 (52.7)	Staff	21	3 (14.2)
			Residents	53	36 (67.9)

Correctional facility					
A	128	49 (38.3)	Staff	82	15 (18.3)
			Inmates	46	34 (73.9)
B	416	61 (14.7)	Staff	210	1 (0.5)
			Inmates	206	60 (29.1)
Meat-processing plant					
A	432	16 (3.7)	Employees	432	16 (3.7)
B	724	5 (0.7)	Employees	724	5 (0.7)
Community sample A	17	8 (47.1)	Known contact	9	2 (22.2)
			No known contact	8	6 (75.0)
Total	2,222	319 (14.4)	NA	NA	NA

*No cases or samples sequenced after June 30, 2020, are included in study. An outbreak is defined as closed if there are no new coronavirus disease cases for 28 days after the onset date of the last case. The outbreak at correctional facility A was considered closed as of July 20; the outbreak at correctional facility B was considered closed as of August 5. The outbreaks at processing plants A and B were considered ongoing as of November 6, 2020. LTCF, long-term care facility; NA, not applicable.

### Single Cluster in LTCFs

**Figure 2:**
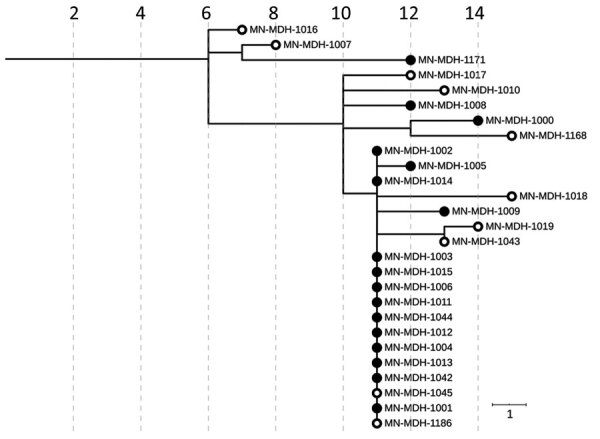
Phylogenetic tree of severe acute respiratory syndrome coronavirus 2 genome sequences associated with long-term care facility A, Minnesota, USA, April 15–June 11, 2020. Solid circles represent sequences in samples from residents; open circles represent sequences from samples from healthcare workers. IQ-TREE (*29*) was used with the general time reversible substitution model for tree generation. Branch lengths were scaled to represent number of single-nucleotide mutations, as shown in the scale. MDH, Minnesota Department of Health.

During the COVID-19 outbreak at LTCF A (*3*), April 15–June 11 (Figure 2), infection was confirmed for 51/77 residents and 38/108 healthcare workers (HCWs) tested after identification of SARS-CoV-2–positive HCWs. Specimens from 17 residents (33.3% of case-patients) and 10 HCWs (26.3% of case-patients) were available for WGS. SARS-CoV-2 viral sequences from these 27 persons were genetically closely related (pangolin lineage B.1.2). Viral genomes from 2 HCWs (MN-MDH-1007 and MN-MDH-1016) sampled on April 30 and 1 resident (MN-MDH-1171) sampled on May 18 at LTCF A did not cluster with each other or the primary outbreak cluster, although all were a part of the broad pangolin lineage B.1.

In LTCF B (*3*) (Appendix Figure 1), during April 29–June 11, SARS-CoV-2 positivity was confirmed for 114 of 182 tested residents and 76 of 233 tested HCWs, after a SARS-CoV-2–positive resident was identified on April 29. All 82 sequenced specimens from this facility, including those from 77 residents (67.5% of case-patients) and 5 HCWs (6.6% of case-patients), were closely related (pangolin lineage B.1.116).

The first COVID-19 case at LTCF C (Appendix Figure 2) was identified on April 24. Four positive HCWs and 3 symptomatic residents were identified by April 30. Throughout May and June, facilitywide testing was implemented; ≈941 residents and staff were tested and 80 SARS-CoV-2–positive residents and 52 SARS-CoV-2–positive staff members were identified. Phylogenetic analysis of the 32 successfully sequenced genomes, including those from 9 residents (10.8% of case-patients) and 23 staff members (41% of case-patients) showed that viruses from 29 of the 32 case-patients were closely related (pangolin lineage B.1.2). Viruses from the remaining 3 case-patients (pangolin lineages B.1 and B.4) were not closely related to each other nor identified with further transmission.

### Two Distinct Clusters in an LTCF

**Figure 3:**
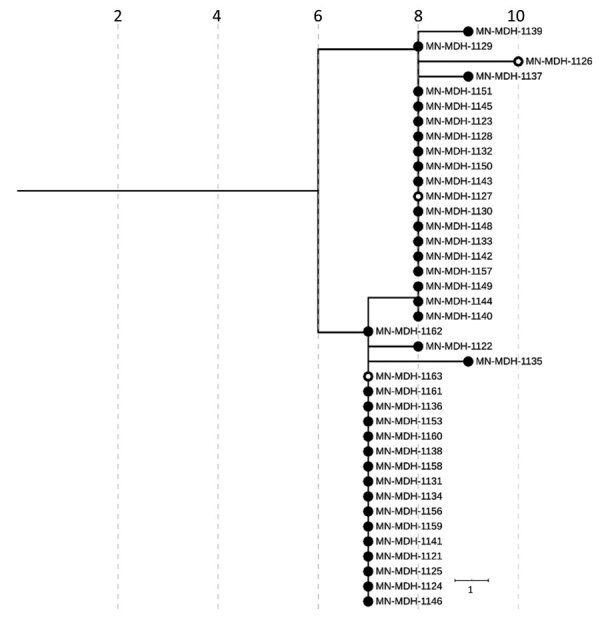
Phylogenetic tree of severe acute respiratory syndrome coronavirus 2 genome sequences associated with long-term care facility D, Minnesota, USA, April 17–May 15, 2020. Filled circles represent sequences taken from residents; open circles represent sequences from healthcare workers. IQ-TREE (*29*) was used with the general time reversible substitution model for tree generation. Branch lengths were scaled to represent number of single-nucleotide mutations, as shown in the scale. MDH, Minnesota Department of Health.

LTCF D (Figure 3) is a 100-bed facility with ≈78 residents and 100 staff, where an outbreak began on April 17, 2020, with a symptomatic HCW. The first cases in residents and staff were identified on April 20, 2020; subsequent testing identified of 53 SARS-CoV-2–positive residents and 21 positive staff members. Although this outbreak was epidemiologically similar to outbreaks at other LTCFs, an analysis of the genetic relatedness among 39 sequenced isolates demonstrated that 2 distinct genetic clusters were in the facility during approximately the same period. In contrast to the outbreaks in LTCFs A, B, and C, viruses from both clusters at LTCF D seemed to circulate simultaneously throughout the facility, each contributing to the outbreak. All sequenced isolates from LTCF D belonged to the broad pangolin lineage B.1.

### Single Cluster in Correctional Facilities

**Figure 4:**
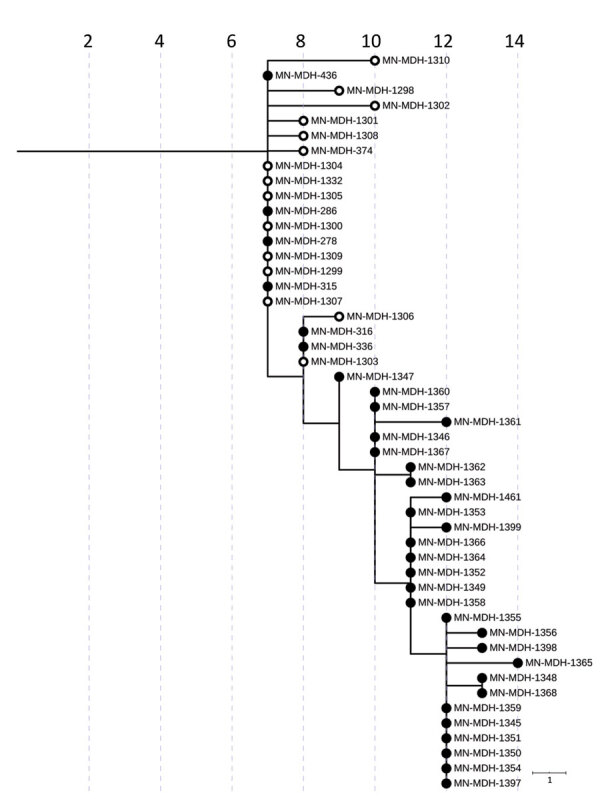
Phylogenetic tree of severe acute respiratory syndrome coronavirus 2 genome sequences associated with correctional facility A, Minnesota, USA, March 25–June 30, 2020. Filled circles represent sequences from samples from inmates, open circles represent sequences from samples from facility staff. IQ-TREE (*29*) was used with the general time reversible substitution model for tree generation. Branch lengths were scaled to represent number of single-nucleotide mutations, as shown in the scale. MDH, Minnesota Department of Health.

In late March 2020, an outbreak of SARS-CoV-2 was identified in correctional facility A (Figure 4). The first identified case-patient was an inmate who became symptomatic and had a positive SARS-CoV-2 test result on March 25. By March 30, a total of 7 confirmed cases and 6 suspected cases among the inmate population were identified. During March 30–April 7, SARS-CoV-2 test results were positive for 15 staff members. Analysis of the genetic relatedness of the virus from 34 inmates (73.9% of case-patients) and 15 staff members (18.3% of case-patients) from correctional facility A were all closely related (pangolin lineage A.1).

In early June 2020, an outbreak was identified in correctional facility B (Appendix Figure 3). The investigation revealed that an employee had symptoms consistent with COVID-19 on May 13, had a positive SARS-CoV-2 test result on May 14, and was subsequently excluded from work and isolated at home. Approximately 2 weeks later, 3 additional case-patients (1 staff member and 2 inmates from the same unit as the index patient) had positive SARS-CoV-2 test results. A point-prevalence survey on June 1 in this unit revealed 63 SARS-CoV-2–positive inmates among the 87 tested. Subsequent facilitywide testing of both staff and inmates identified cases in other units, 83 new cases in inmates and 1 new case in a staff member, identified among the ≈2,200 persons tested. Test results were ultimately positive for 210 staff members and 206 inmates during this outbreak. Phylogenetic analysis of viruses from this outbreak among the 1 staff member (0.5% of staff case-patients) and 60 inmates (29.1% of inmate case-patients) at correctional facility B shows that all viruses were closely related (pangolin lineage B.1.2) and genetically identical to, or plausibly descended from, the sequence of SARS-CoV-2 from the index case-patient.

### Linking LTCF C with Correctional Facility B

**Figure 5:**
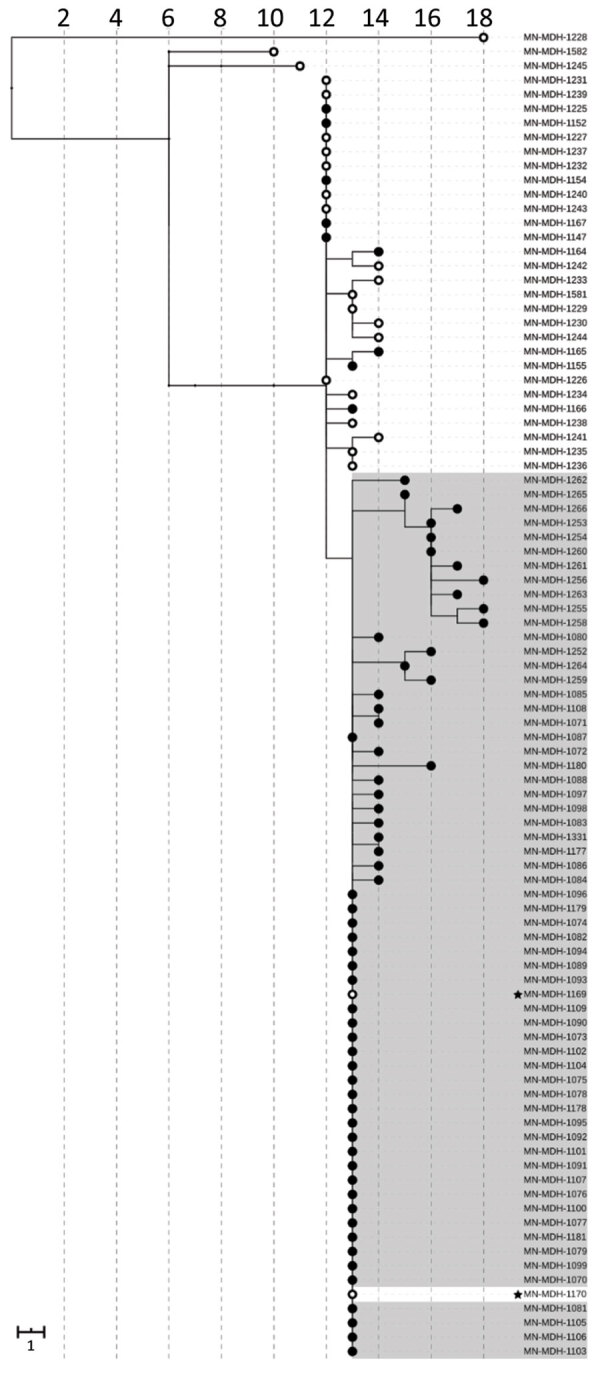
Phylogenetic tree of severe acute respiratory syndrome coronavirus 2 genome sequences associated with long-term care facility C and correctional facility B, Minnesota, US, April–June 2020. Filled circles represent sequences from samples from inmates or residents; open circles represent sequences from facility staff or healthcare workers. Sequences from long-term care facility C are shown on a white background; sequences from correctional facility B, on a gray background. Sequences from 2 household contacts are noted with stars. IQ-TREE (*29*) was used with the general time reversible substitution model for tree generation. Branch lengths were scaled to represent number of single-nucleotide mutations, as shown in the scale. MDH, Minnesota Department of Health.

During the epidemiologic investigation at LTCF C, we learned that an HCW at LTCF C was a household contact of a correctional facility B employee. Both persons became symptomatic at the same time, and both subsequently had positive test results in mid-May. SARS-CoV-2 genome sequences recovered from these 2 household contacts were identical to each other and to the genomic sequences recovered from 32 inmates at correctional facility B (Figure 5). In addition, this genomic sequence differs by only a single mutation (G5617T) from isolates sequenced from 13 case-patients at LTCF C.

### Multiple Clusters in Meat-Processing Plants

**Figure 6:**
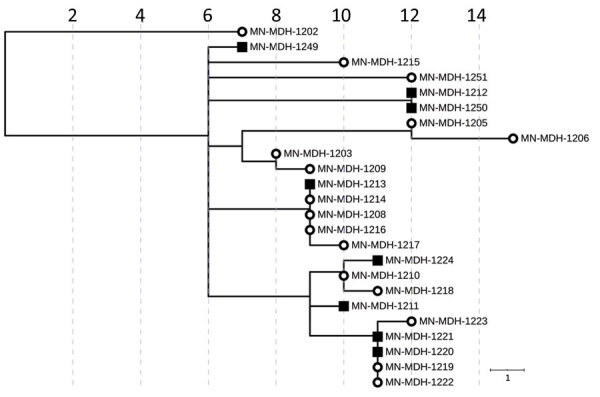
Phylogenetic tree of SARS-CoV-2 genome sequences associated with meat-processing plant A and the surrounding community, Minnesota, USA, March 15–June 30, 2020. Open circles represent sequences from samples from staff at processing plant A; squares represent sequences from samples from persons in the surrounding community. IQ-TREE (*29*) was used with the general time reversible substitution model for tree generation. Branch lengths were scaled to represent number of single-nucleotide mutations, as shown in the scale. MDH, Minnesota Department of Health.

In early April 2020, an outbreak was detected at processing plant A (Figure 6), a large primary and secondary meat processor. This outbreak continued for several weeks until mid-May, when the number of cases among workers began to increase rapidly. During March 15–July 1, a total of 446 persons with confirmed cases who reported working at processing plant A, including 4 (1%) case-patients with positive test results in March (management and office staff), 5 (1%) in April, 211 (47%) in May, and 226 (51%) in June. Of the 16 samples (3.7% of case-patients) sequenced during March 15–June 3, at least 6 clusters or single cases were unrelated. Although most genomes sequenced from processing plant B belonged to pangolin lineages B.1, B1.2, B.1.26, one early case is genetically quite different (pangolin lineage A.1). An interview confirmed that this early case-patient had traveled out of the state during the exposure period (14 days before symptom onset).

During May 15–June 1, we sequenced samples obtained from 8 case-patients in the county where processing plant A is located (community samples A). From these 8 samples, we identified 5 clusters. Of the 8 samples, 5 were closely related with 3 clusters from processing plant A, while the remaining 3 samples formed 2 distinct clusters. Of the 5 sequences from community samples A that clustered with sequences from processing plant A, 4 had sequences that were identical to sequences from processing plant A, and all 4 persons had no known contact with a verified case-patient.

In mid-April 2020, an outbreak was identified among employees at processing plant B (Appendix Figure 4), another large meat-processing plant. By May 1, a total of 649 cases among workers at processing plant B were confirmed. Sequencing of the 5 available samples from processing plant B (0.7% of cases) identified 1 cluster and 2 single genomes, all belonging to pangolin lineage B.1.

## Discussion

WGS identified 3 primary patterns of genetic relatedness among cases in various outbreak settings: outbreaks in which cases were part of 1 genetically related cluster; an outbreak with 2 unique clusters of cases, each contributing to the outbreak during the same period; and outbreaks for which multiple genetically distinct sequences were present. Phylogenetic analyses of the viral sequences from available specimens (Appendix Table 1) associated with outbreaks in LTCFs A, B, and C were all consistent with at >1 primary cluster affecting each facility, suggesting that a single introduction of SARS-CoV-2 into a facility can result in a widespread outbreak. This finding is similar to previously reported findings, in which WGS has evidenced rapid spread in high-density settings as opposed to multiple introductions contributing to the outbreak (*20*). Cases from LTCF D, in contrast, formed 2 distinct genetic clusters, 1 consisting of 17 related samples and the other consisting of 22 samples. This finding is consistent with a potential scenario in which there were 2 separate, independent introductions into the facility and subsequent parallel intrafacility spread of each individually distinct sequence.

Phylogenetic analysis conducted for LTCFs A and C also demonstrated outlier SARS-CoV-2 viral sequences that were not genetically closely related to the primary cluster in each facility. This finding suggests community-acquired infection and subsequent introduction of SARS-CoV-2 into the facility (*3*). Two of the 3 outlier case-patients at LTCF C had positive test results >1 month after the first identified case. Similarly, 2 of the 3 outlier case-patients identified at LTCF A were identified 10 days after the first identified case-patient, and the third had a positive test result 28 days later. It is not possible to determine whether these introductions of distinct genetic sequences resulted in additional spread, given that WGS characterization was not performed on all positive samples in each facility and not all HCWs or residents were tested. However, the timing of the identification of these outlier cases after the date of the first identified primary case suggests that mitigation strategies implemented after the initial identification of the outbreak, including cohorting strategies, infection prevention and control measures, and correct use of personal protective equipment (PPE), may have effectively prevented intrafacility transmission of these late outlier cases, as has been reported (*3*,*21**,**22*).

WGS identified a different genetic landscape in meat-processing plants, in which several distinct sequences contributed to the facility outbreak. This finding is despite sequencing of only 2.5% of SARS-CoV-2–positive samples from the processing plants, suggesting that increased sequencing may have identified even greater genetic diversity. In addition, several genomes identified at processing plant A were either identical or closely related to genomes in the surrounding community (community samples A). Of the 8 sequenced community samples (community sample A), 6 were from persons with no known epidemiologic link to a case-patient at processing plant A, strongly suggesting an unrecognized connection. The benefit of WGS for identifying previously unrecognized transmission patterns has been established (*20*,*32*). Although no definitive conclusions can be made regarding the direction of transmission, WGS provided strong evidence of worker/community member spread; hypothesized factors potentially contributing to this transmission pattern are communal housing, multigenerational families, and group transportation.

WGS has contributed to improved knowledge of an outbreak after retrospective analysis (G.K. Moreno et al., unpub. data, https://doi.org/10.1101/2020.07.09.20149104) (*3**,**20**,**21*), justification for specific public health measures (*21**,**22*), and added insight to transmission patterns in high-risk settings. Our work further supports use of WGS in these situations while identifying several additional public health implications. WGS has demonstrated that outbreaks in LTCFs and correctional facilities can result from a single introduction. Continued vigilance, including facilitywide staff screening and subsequent exclusion of symptomatic HCWs or staff and those with known or suspected contacts, is imperative. WGS has demonstrated extensive intrafacility spread; closely related sequences comprise all or most cases contributing to the outbreak. Measures such as infection prevention and control, consistent and correct use of PPE, cohorting of known positive residents, and exclusion of positive HCWs must be maintained. WGS has also illuminated the transmission patterns in processing plants, including the multiple introductions identified through the multiple genetically distinct sequences identified and the related community strains. WGS has illustrated the need for community-level mitigation to prevent introductions in high-density worksites, including accessible communitywide testing, housing and transportation strategies, and facility-level measures to prevent unintended introduction into the workplace.

The first limitation of this study is that only a subset of specimens were available for sequencing because of different laboratory specimen retention policies. For example, at LTCF B, samples from only 5 staff members were available for sequencing. Similarly, in meat-processing plant B, only 5 samples were available because of a clinical testing laboratory protocol that resulted in the discarding of samples after ≈7 days. In addition, not all available samples could be successfully sequenced, primarily because of degraded quality or low concentrations of viral RNA.

Another limitation is that not all staff and employees at the LTCFs, correctional facilities, and processing plants agreed to be tested. Because of the incomplete genomic picture at each setting, definitive conclusions about single introductions in LTCFs A and D are speculative, and these individual introductions may have resulted in some virus transmission that was not identified in the study.

Last, we were not able to present sociodemographic data such as race or ethnicity associated with these outbreaks because of limitations in the case investigation process and incomplete case data. This limitation is particularly relevant because of the disproportionate effect of COVID-19 on those who are Black, indigenous, or other persons of color. Because those populations disproportionately experience incarceration and a high proportion of meat-processing plant employees are persons from immigrant communities, these settings can serve to amplify racial and ethnic health disparities related to COVID-19.

LTCFs, correctional facilities, and high-density workplace settings have many factors that are hypothesized to contribute to rapid transmission of SARS-CoV-2. These factors include insufficient resources and training in infection prevention and control, difficulties implementing social distancing because of close habitation or work environment, and delayed case detection and access to care (*8*,*11*,*33*). WGS results have demonstrated that many outbreaks in Minnesota were caused by single introductions of SARS-CoV-2, highlighting the value of consistent and correct PPE use, rigorous and systematic infection prevention and control, environmental control measures, and systematic testing of residents and staff to identify asymptomatic infected persons. As this pandemic continues, community mitigation strategies and strong enforcement of policies to reduce the risk of introducing SARS-CoV-2 virus into congregate settings are more crucial than ever. Similarly, infection prevention and control and aggressive containment practices are vital for mitigating the spread of SARS-CoV-2 after its introduction into a facility. WGS can be a useful tool for supplementing epidemiologic information and examining the role of facility and community factors contributing to SARS-COV-2 outbreaks in high-risk settings.

Appendix Supplemental results from study of transmission dynamics of severe acute respiratory syndrome coronavirus 2 in high-density settings, Minnesota, USA, March–June 2020.
