# Supplementary material for: Transmission Dynamics of Severe Acute Respiratory Syndrome Coronavirus 2 in High-Density Settings, Minnesota, USA, March–June 2020
Source: Emerg Infect Dis. 2021 Aug;27(8):2052–63. doi: 10.3201/eid2708.204838 (PMC8314815; doi:10.3201/eid2708.204838)
Supplement: Appendix — Supplemental results from study of transmission dynamics of severe acute respiratory syndrome coronavirus 2 in high-density settings, Minnesota, USA, March–June 2020. [file 20-4838-Techapp-s1.pdf]

Article DOI: https://doi.org/10.3201/eid2708.204838

Transmission Dynamics of Severe Acute Respiratory Syndrome Coronavirus 2 in High-Density Settings, Minnesota, USA, March–June 2020

Appendix

Appendix Table. GISAID accession numbers for all sequenced samples from outbreak settings*

Outbreak	Genome	GISAID Accession
Community Samples A	MN-MDH-1211	EPI_ISL_477300
Community Samples A	MN-MDH-1212	EPI_ISL_477301
Community Samples A	MN-MDH-1213	EPI_ISL_477302
Community Samples A	MN-MDH-1220	EPI_ISL_477309
Community Samples A	MN-MDH-1221	EPI_ISL_477310
Community Samples A	MN-MDH-1224	EPI_ISL_481242
Community Samples A	MN-MDH-1249	EPI_ISL_482970
Community Samples A	MN-MDH-1250	EPI_ISL_482971
Correctional Facility A	MN-MDH-286	EPI_ISL_437362
Correctional Facility A	MN-MDH-315	EPI_ISL_437363
Correctional Facility A	MN-MDH-336	EPI_ISL_437364
Correctional Facility A	MN-MDH-374	EPI_ISL_437366
Correctional Facility A	MN-MDH-436	EPI_ISL_437373
Correctional Facility A	MN-MDH-278	EPI_ISL_450752
Correctional Facility A	MN-MDH-316	EPI_ISL_450774
Correctional Facility A	MN-MDH-1298	EPI_ISL_495597
Correctional Facility A	MN-MDH-1299	EPI_ISL_495598
Correctional Facility A	MN-MDH-1300	EPI_ISL_495599
Correctional Facility A	MN-MDH-1301	EPI_ISL_495600
Correctional Facility A	MN-MDH-1302	EPI_ISL_495601
Correctional Facility A	MN-MDH-1303	EPI_ISL_495602
Correctional Facility A	MN-MDH-1304	EPI_ISL_495603
Correctional Facility A	MN-MDH-1305	EPI_ISL_495604
Correctional Facility A	MN-MDH-1306	EPI_ISL_495605
Correctional Facility A	MN-MDH-1307	EPI_ISL_495606
Correctional Facility A	MN-MDH-1308	EPI_ISL_495607
Correctional Facility A	MN-MDH-1309	EPI_ISL_495608
Correctional Facility A	MN-MDH-1310	EPI_ISL_495609
Correctional Facility A	MN-MDH-1332	EPI_ISL_496918
Correctional Facility A	MN-MDH-1345	EPI_ISL_507934
Correctional Facility A	MN-MDH-1346	EPI_ISL_507935
Correctional Facility A	MN-MDH-1347	EPI_ISL_507936
Correctional Facility A	MN-MDH-1348	EPI_ISL_507937
Correctional Facility A	MN-MDH-1349	EPI_ISL_507938
Correctional Facility A	MN-MDH-1350	EPI_ISL_507939
Correctional Facility A	MN-MDH-1351	EPI_ISL_507940
Correctional Facility A	MN-MDH-1352	EPI_ISL_507941
Correctional Facility A	MN-MDH-1353	EPI_ISL_507942
Correctional Facility A	MN-MDH-1354	EPI_ISL_507943
Correctional Facility A	MN-MDH-1355	EPI_ISL_507944
Correctional Facility A	MN-MDH-1356	EPI_ISL_507945
Correctional Facility A	MN-MDH-1357	EPI_ISL_507946
Correctional Facility A	MN-MDH-1358	EPI_ISL_507947
Correctional Facility A	MN-MDH-1359	EPI_ISL_507948
Correctional Facility A	MN-MDH-1360	EPI_ISL_507949
Correctional Facility A	MN-MDH-1361	EPI_ISL_507950
Correctional Facility A	MN-MDH-1362	EPI_ISL_507951
Correctional Facility A	MN-MDH-1363	EPI_ISL_507952

Outbreak	Genome	GISAID Accession
Correctional Facility A	MN-MDH-1364	EPI_ISL_515262
Correctional Facility A	MN-MDH-1365	EPI_ISL_507953
Correctional Facility A	MN-MDH-1366	EPI_ISL_507954
Correctional Facility A	MN-MDH-1367	EPI_ISL_507955
Correctional Facility A	MN-MDH-1368	EPI_ISL_507956
Correctional Facility A	MN-MDH-1397	EPI_ISL_514616
Correctional Facility A	MN-MDH-1398	EPI_ISL_514617
Correctional Facility A	MN-MDH-1399	EPI_ISL_514618
Correctional Facility A	MN-MDH-1461	EPI_ISL_518855
Correctional Facility B	MN-MDH-1070	EPI_ISL_470750
Correctional Facility B	MN-MDH-1071	EPI_ISL_470751
Correctional Facility B	MN-MDH-1072	EPI_ISL_470752
Correctional Facility B	MN-MDH-1073	EPI_ISL_470753
Correctional Facility B	MN-MDH-1074	EPI_ISL_470754
Correctional Facility B	MN-MDH-1075	EPI_ISL_470755
Correctional Facility B	MN-MDH-1076	EPI_ISL_470756
Correctional Facility B	MN-MDH-1077	EPI_ISL_470757
Correctional Facility B	MN-MDH-1078	EPI_ISL_470758
Correctional Facility B	MN-MDH-1079	EPI_ISL_470759
Correctional Facility B	MN-MDH-1080	EPI_ISL_470760
Correctional Facility B	MN-MDH-1081	EPI_ISL_470761
Correctional Facility B	MN-MDH-1082	EPI_ISL_470762
Correctional Facility B	MN-MDH-1083	EPI_ISL_470763
Correctional Facility B	MN-MDH-1084	EPI_ISL_470764
Correctional Facility B	MN-MDH-1085	EPI_ISL_470765
Correctional Facility B	MN-MDH-1086	EPI_ISL_470766
Correctional Facility B	MN-MDH-1087	EPI_ISL_470767
Correctional Facility B	MN-MDH-1088	EPI_ISL_470768
Correctional Facility B	MN-MDH-1089	EPI_ISL_470769
Correctional Facility B	MN-MDH-1090	EPI_ISL_470770
Correctional Facility B	MN-MDH-1091	EPI_ISL_470771
Correctional Facility B	MN-MDH-1092	EPI_ISL_470772
Correctional Facility B	MN-MDH-1093	EPI_ISL_470773
Correctional Facility B	MN-MDH-1094	EPI_ISL_470774
Correctional Facility B	MN-MDH-1095	EPI_ISL_470775
Correctional Facility B	MN-MDH-1096	EPI_ISL_470776
Correctional Facility B	MN-MDH-1097	EPI_ISL_470777
Correctional Facility B	MN-MDH-1098	EPI_ISL_470778
Correctional Facility B	MN-MDH-1099	EPI_ISL_470779
Correctional Facility B	MN-MDH-1100	EPI_ISL_470780
Correctional Facility B	MN-MDH-1101	EPI_ISL_470781
Correctional Facility B	MN-MDH-1102	EPI_ISL_470782
Correctional Facility B	MN-MDH-1103	EPI_ISL_470783
Correctional Facility B	MN-MDH-1104	EPI_ISL_470784
Correctional Facility B	MN-MDH-1105	EPI_ISL_470785
Correctional Facility B	MN-MDH-1106	EPI_ISL_470786
Correctional Facility B	MN-MDH-1107	EPI_ISL_470787
Correctional Facility B	MN-MDH-1108	EPI_ISL_470788
Correctional Facility B	MN-MDH-1109	EPI_ISL_470789
Correctional Facility B	MN-MDH-1169	EPI_ISL_476754
Correctional Facility B	MN-MDH-1177	EPI_ISL_476762
Correctional Facility B	MN-MDH-1178	EPI_ISL_476763
Correctional Facility B	MN-MDH-1179	EPI_ISL_476764
Correctional Facility B	MN-MDH-1180	EPI_ISL_476765
Correctional Facility B	MN-MDH-1181	EPI_ISL_476766
Correctional Facility B	MN-MDH-1252	EPI_ISL_482973
Correctional Facility B	MN-MDH-1253	EPI_ISL_482974
Correctional Facility B	MN-MDH-1254	EPI_ISL_482975
Correctional Facility B	MN-MDH-1255	EPI_ISL_482976
Correctional Facility B	MN-MDH-1256	EPI_ISL_482977
Correctional Facility B	MN-MDH-1258	EPI_ISL_482979
Correctional Facility B	MN-MDH-1259	EPI_ISL_482980
Correctional Facility B	MN-MDH-1260	EPI_ISL_482981
Correctional Facility B	MN-MDH-1261	EPI_ISL_482982
Correctional Facility B	MN-MDH-1262	EPI_ISL_482983
Correctional Facility B	MN-MDH-1263	EPI_ISL_482984
Correctional Facility B	MN-MDH-1264	EPI_ISL_482985
Correctional Facility B	MN-MDH-1265	EPI_ISL_482986
Correctional Facility B	MN-MDH-1266	EPI_ISL_482987

Outbreak	Genome	GISAID Accession
Correctional Facility B	MN-MDH-1331	EPI_ISL_496917
LTCF A	MN-MDH-1000	EPI_ISL_462845
LTCF A	MN-MDH-1001	EPI_ISL_462846
LTCF A	MN-MDH-1002	EPI_ISL_462847
LTCF A	MN-MDH-1003	EPI_ISL_462848
LTCF A	MN-MDH-1004	EPI_ISL_462849
LTCF A	MN-MDH-1005	EPI_ISL_462850
LTCF A	MN-MDH-1006	EPI_ISL_462851
LTCF A	MN-MDH-1007	EPI_ISL_462852
LTCF A	MN-MDH-1008	EPI_ISL_462853
LTCF A	MN-MDH-1009	EPI_ISL_462854
LTCF A	MN-MDH-1010	EPI_ISL_462855
LTCF A	MN-MDH-1011	EPI_ISL_462856
LTCF A	MN-MDH-1012	EPI_ISL_462857
LTCF A	MN-MDH-1013	EPI_ISL_462858
LTCF A	MN-MDH-1014	EPI_ISL_462859
LTCF A	MN-MDH-1015	EPI_ISL_462860
LTCF A	MN-MDH-1016	EPI_ISL_462861
LTCF A	MN-MDH-1017	EPI_ISL_462862
LTCF A	MN-MDH-1018	EPI_ISL_462863
LTCF A	MN-MDH-1019	EPI_ISL_462864
LTCF A	MN-MDH-1042	EPI_ISL_462887
LTCF A	MN-MDH-1043	EPI_ISL_462888
LTCF A	MN-MDH-1044	EPI_ISL_462889
LTCF A	MN-MDH-1045	EPI_ISL_462890
LTCF A	MN-MDH-1168	EPI_ISL_476753
LTCF A	MN-MDH-1171	EPI_ISL_476756
LTCF A	MN-MDH-1186	EPI_ISL_477276
LTCF B	MN-MDH-1020	EPI_ISL_462865
LTCF B	MN-MDH-1021	EPI_ISL_462866
LTCF B	MN-MDH-1022	EPI_ISL_462867
LTCF B	MN-MDH-1023	EPI_ISL_462868
LTCF B	MN-MDH-1024	EPI_ISL_462869
LTCF B	MN-MDH-1025	EPI_ISL_462870
LTCF B	MN-MDH-1026	EPI_ISL_462871
LTCF B	MN-MDH-1027	EPI_ISL_462872
LTCF B	MN-MDH-1028	EPI_ISL_462873
LTCF B	MN-MDH-1029	EPI_ISL_462874
LTCF B	MN-MDH-1030	EPI_ISL_462875
LTCF B	MN-MDH-1031	EPI_ISL_462876
LTCF B	MN-MDH-1032	EPI_ISL_462877
LTCF B	MN-MDH-1033	EPI_ISL_462878
LTCF B	MN-MDH-1034	EPI_ISL_462879
LTCF B	MN-MDH-1035	EPI_ISL_462880
LTCF B	MN-MDH-1036	EPI_ISL_462881
LTCF B	MN-MDH-1037	EPI_ISL_462882
LTCF B	MN-MDH-1038	EPI_ISL_462883
LTCF B	MN-MDH-1039	EPI_ISL_462884
LTCF B	MN-MDH-1040	EPI_ISL_462885
LTCF B	MN-MDH-1041	EPI_ISL_462886
LTCF B	MN-MDH-1046	EPI_ISL_462891
LTCF B	MN-MDH-1047	EPI_ISL_462892
LTCF B	MN-MDH-1048	EPI_ISL_462893
LTCF B	MN-MDH-1049	EPI_ISL_462894
LTCF B	MN-MDH-1050	EPI_ISL_462895
LTCF B	MN-MDH-1051	EPI_ISL_462896
LTCF B	MN-MDH-1052	EPI_ISL_462897
LTCF B	MN-MDH-1053	EPI_ISL_462898
LTCF B	MN-MDH-1054	EPI_ISL_462899
LTCF B	MN-MDH-1055	EPI_ISL_462900
LTCF B	MN-MDH-1056	EPI_ISL_462901
LTCF B	MN-MDH-1057	EPI_ISL_462902
LTCF B	MN-MDH-1058	EPI_ISL_462903
LTCF B	MN-MDH-1059	EPI_ISL_462904
LTCF B	MN-MDH-1060	EPI_ISL_462905
LTCF B	MN-MDH-1061	EPI_ISL_462906
LTCF B	MN-MDH-1062	EPI_ISL_462907
LTCF B	MN-MDH-1063	EPI_ISL_462908
LTCF B	MN-MDH-1064	EPI_ISL_462909

Outbreak	Genome	GISAID Accession
LTCF B	MN-MDH-1065	EPI_ISL_462910
LTCF B	MN-MDH-1066	EPI_ISL_462911
LTCF B	MN-MDH-1067	EPI_ISL_470747
LTCF B	MN-MDH-1068	EPI_ISL_470748
LTCF B	MN-MDH-1069	EPI_ISL_470749
LTCF B	MN-MDH-1110	EPI_ISL_470790
LTCF B	MN-MDH-1111	EPI_ISL_470791
LTCF B	MN-MDH-1112	EPI_ISL_470792
LTCF B	MN-MDH-1113	EPI_ISL_470793
LTCF B	MN-MDH-1114	EPI_ISL_470794
LTCF B	MN-MDH-1115	EPI_ISL_470795
LTCF B	MN-MDH-1116	EPI_ISL_470796
LTCF B	MN-MDH-1117	EPI_ISL_470797
LTCF B	MN-MDH-1118	EPI_ISL_470798
LTCF B	MN-MDH-1119	EPI_ISL_470799
LTCF B	MN-MDH-1120	EPI_ISL_470800
LTCF B	MN-MDH-1172	EPI_ISL_476757
LTCF B	MN-MDH-1173	EPI_ISL_476758
LTCF B	MN-MDH-1174	EPI_ISL_476759
LTCF B	MN-MDH-1175	EPI_ISL_476760
LTCF B	MN-MDH-1176	EPI_ISL_476761
LTCF B	MN-MDH-1182	EPI_ISL_477272
LTCF B	MN-MDH-1183	EPI_ISL_477273
LTCF B	MN-MDH-1184	EPI_ISL_477274
LTCF B	MN-MDH-1185	EPI_ISL_477275
LTCF B	MN-MDH-1187	EPI_ISL_477277
LTCF B	MN-MDH-1188	EPI_ISL_477278
LTCF B	MN-MDH-1189	EPI_ISL_477279
LTCF B	MN-MDH-1190	EPI_ISL_477280
LTCF B	MN-MDH-1191	EPI_ISL_477281
LTCF B	MN-MDH-1192	EPI_ISL_477282
LTCF B	MN-MDH-1193	EPI_ISL_477283
LTCF B	MN-MDH-1194	EPI_ISL_477284
LTCF B	MN-MDH-1195	EPI_ISL_477285
LTCF B	MN-MDH-1196	EPI_ISL_477286
LTCF B	MN-MDH-1197	EPI_ISL_477287
LTCF B	MN-MDH-1198	EPI_ISL_477288
LTCF B	MN-MDH-1199	EPI_ISL_477289
LTCF B	MN-MDH-1200	EPI_ISL_481241
LTCF B	MN-MDH-1201	EPI_ISL_477290
LTCF B	MN-MDH-1330	EPI_ISL_496916
LTCF C	MN-MDH-1147	EPI_ISL_476732
LTCF C	MN-MDH-1152	EPI_ISL_476737
LTCF C	MN-MDH-1154	EPI_ISL_476739
LTCF C	MN-MDH-1155	EPI_ISL_476740
LTCF C	MN-MDH-1164	EPI_ISL_476749
LTCF C	MN-MDH-1165	EPI_ISL_476750
LTCF C	MN-MDH-1166	EPI_ISL_476751
LTCF C	MN-MDH-1167	EPI_ISL_476752
LTCF C	MN-MDH-1170	EPI_ISL_476755
LTCF C	MN-MDH-1225	EPI_ISL_482946
LTCF C	MN-MDH-1226	EPI_ISL_482947
LTCF C	MN-MDH-1227	EPI_ISL_482948
LTCF C	MN-MDH-1228	EPI_ISL_482949
LTCF C	MN-MDH-1229	EPI_ISL_482950
LTCF C	MN-MDH-1230	EPI_ISL_482951
LTCF C	MN-MDH-1231	EPI_ISL_482952
LTCF C	MN-MDH-1232	EPI_ISL_482953
LTCF C	MN-MDH-1233	EPI_ISL_482954
LTCF C	MN-MDH-1234	EPI_ISL_482955
LTCF C	MN-MDH-1235	EPI_ISL_482956
LTCF C	MN-MDH-1236	EPI_ISL_482957
LTCF C	MN-MDH-1237	EPI_ISL_482958
LTCF C	MN-MDH-1238	EPI_ISL_482959
LTCF C	MN-MDH-1239	EPI_ISL_482960
LTCF C	MN-MDH-1240	EPI_ISL_482961
LTCF C	MN-MDH-1241	EPI_ISL_482962
LTCF C	MN-MDH-1242	EPI_ISL_482963
LTCF C	MN-MDH-1243	EPI_ISL_482964

Outbreak	Genome	GISAID Accession
LTCF C	MN-MDH-1244	EPI_ISL_482965
LTCF C	MN-MDH-1245	EPI_ISL_482966
LTCF C	MN-MDH-1581	EPI_ISL_530177
LTCF C	MN-MDH-1582	EPI_ISL_530178
LTCF D	MN-MDH-1121	EPI_ISL_476706
LTCF D	MN-MDH-1122	EPI_ISL_476707
LTCF D	MN-MDH-1123	EPI_ISL_476708
LTCF D	MN-MDH-1124	EPI_ISL_476709
LTCF D	MN-MDH-1125	EPI_ISL_476710
LTCF D	MN-MDH-1126	EPI_ISL_476711
LTCF D	MN-MDH-1127	EPI_ISL_476712
LTCF D	MN-MDH-1128	EPI_ISL_476713
LTCF D	MN-MDH-1129	EPI_ISL_476714
LTCF D	MN-MDH-1130	EPI_ISL_476715
LTCF D	MN-MDH-1131	EPI_ISL_476716
LTCF D	MN-MDH-1132	EPI_ISL_476717
LTCF D	MN-MDH-1133	EPI_ISL_476718
LTCF D	MN-MDH-1134	EPI_ISL_476719
LTCF D	MN-MDH-1135	EPI_ISL_476720
LTCF D	MN-MDH-1136	EPI_ISL_476721
LTCF D	MN-MDH-1137	EPI_ISL_476722
LTCF D	MN-MDH-1138	EPI_ISL_476723
LTCF D	MN-MDH-1139	EPI_ISL_476724
LTCF D	MN-MDH-1140	EPI_ISL_476725
LTCF D	MN-MDH-1141	EPI_ISL_476726
LTCF D	MN-MDH-1142	EPI_ISL_476727
LTCF D	MN-MDH-1143	EPI_ISL_476728
LTCF D	MN-MDH-1144	EPI_ISL_476729
LTCF D	MN-MDH-1145	EPI_ISL_476730
LTCF D	MN-MDH-1146	EPI_ISL_476731
LTCF D	MN-MDH-1148	EPI_ISL_476733
LTCF D	MN-MDH-1149	EPI_ISL_476734
LTCF D	MN-MDH-1150	EPI_ISL_476735
LTCF D	MN-MDH-1151	EPI_ISL_476736
LTCF D	MN-MDH-1153	EPI_ISL_476738
LTCF D	MN-MDH-1156	EPI_ISL_476741
LTCF D	MN-MDH-1157	EPI_ISL_476742
LTCF D	MN-MDH-1158	EPI_ISL_476743
LTCF D	MN-MDH-1159	EPI_ISL_476744
LTCF D	MN-MDH-1160	EPI_ISL_476745
LTCF D	MN-MDH-1161	EPI_ISL_476746
LTCF D	MN-MDH-1162	EPI_ISL_476747
LTCF D	MN-MDH-1163	EPI_ISL_476748
Processing Plant A	MN-MDH-1202	EPI_ISL_477291
Processing Plant A	MN-MDH-1203	EPI_ISL_477292
Processing Plant A	MN-MDH-1205	EPI_ISL_477294
Processing Plant A	MN-MDH-1206	EPI_ISL_477295
Processing Plant A	MN-MDH-1208	EPI_ISL_477297
Processing Plant A	MN-MDH-1209	EPI_ISL_477298
Processing Plant A	MN-MDH-1210	EPI_ISL_477299
Processing Plant A	MN-MDH-1214	EPI_ISL_477303
Processing Plant A	MN-MDH-1215	EPI_ISL_477304
Processing Plant A	MN-MDH-1216	EPI_ISL_477305
Processing Plant A	MN-MDH-1217	EPI_ISL_477306
Processing Plant A	MN-MDH-1218	EPI_ISL_477307
Processing Plant A	MN-MDH-1219	EPI_ISL_477308
Processing Plant A	MN-MDH-1222	EPI_ISL_477311
Processing Plant A	MN-MDH-1223	EPI_ISL_477312
Processing Plant A	MN-MDH-1251	EPI_ISL_482972
Processing Plant B	MN-MDH-1204	EPI_ISL_477293
Processing Plant B	MN-MDH-1207	EPI_ISL_477296
Processing Plant B	MN-MDH-1246	EPI_ISL_482967
Processing Plant B	MN-MDH-1247	EPI_ISL_482968
Processing Plant B	MN-MDH-1248	EPI_ISL_482969

*GISAID, http://www.gisaid.org.

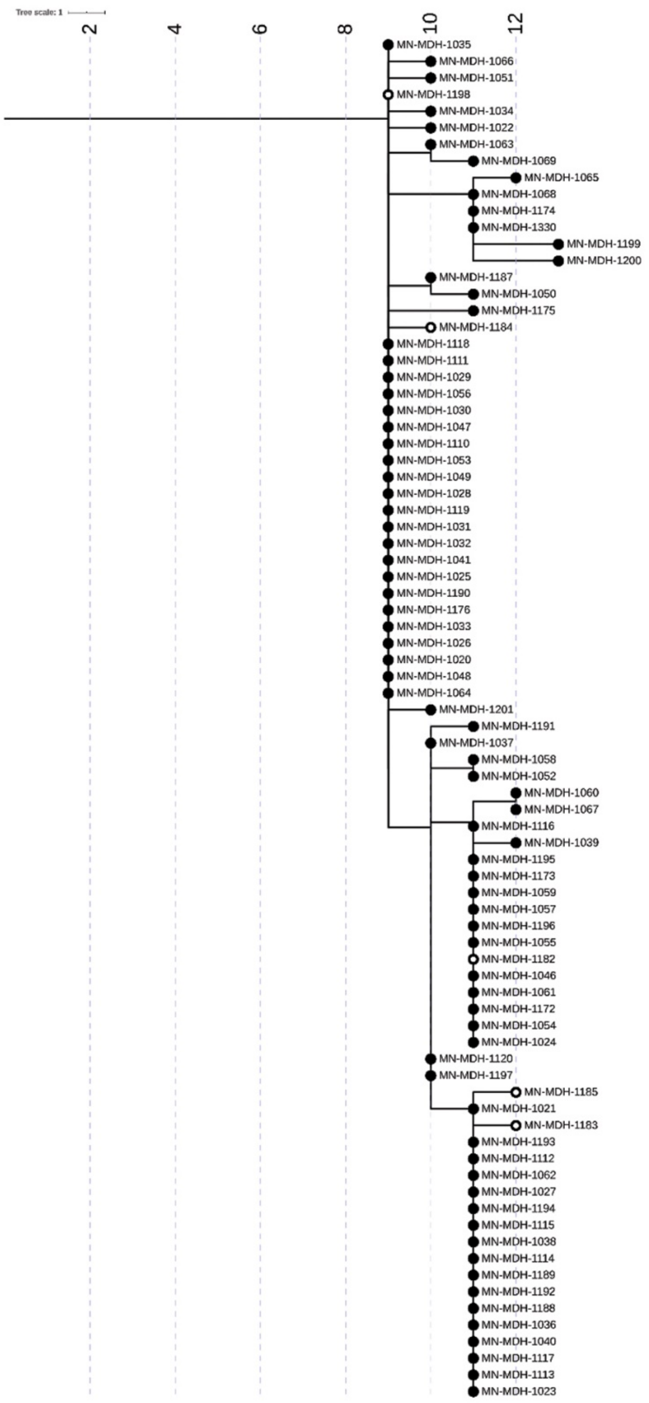

Appendix Figure 1. Phylogenetic tree of SARS-CoV-2 genome sequences associated with LTCF B from April 29 to June 11, 2020. Filled circles represent sequences taken from residents, open circles represent sequences from healthcare workers. IQ-TREE was used with the general time reversible substitution model for tree generation. Branch lengths were scaled to represent number of single nucleotide mutations as shown in the scale key.

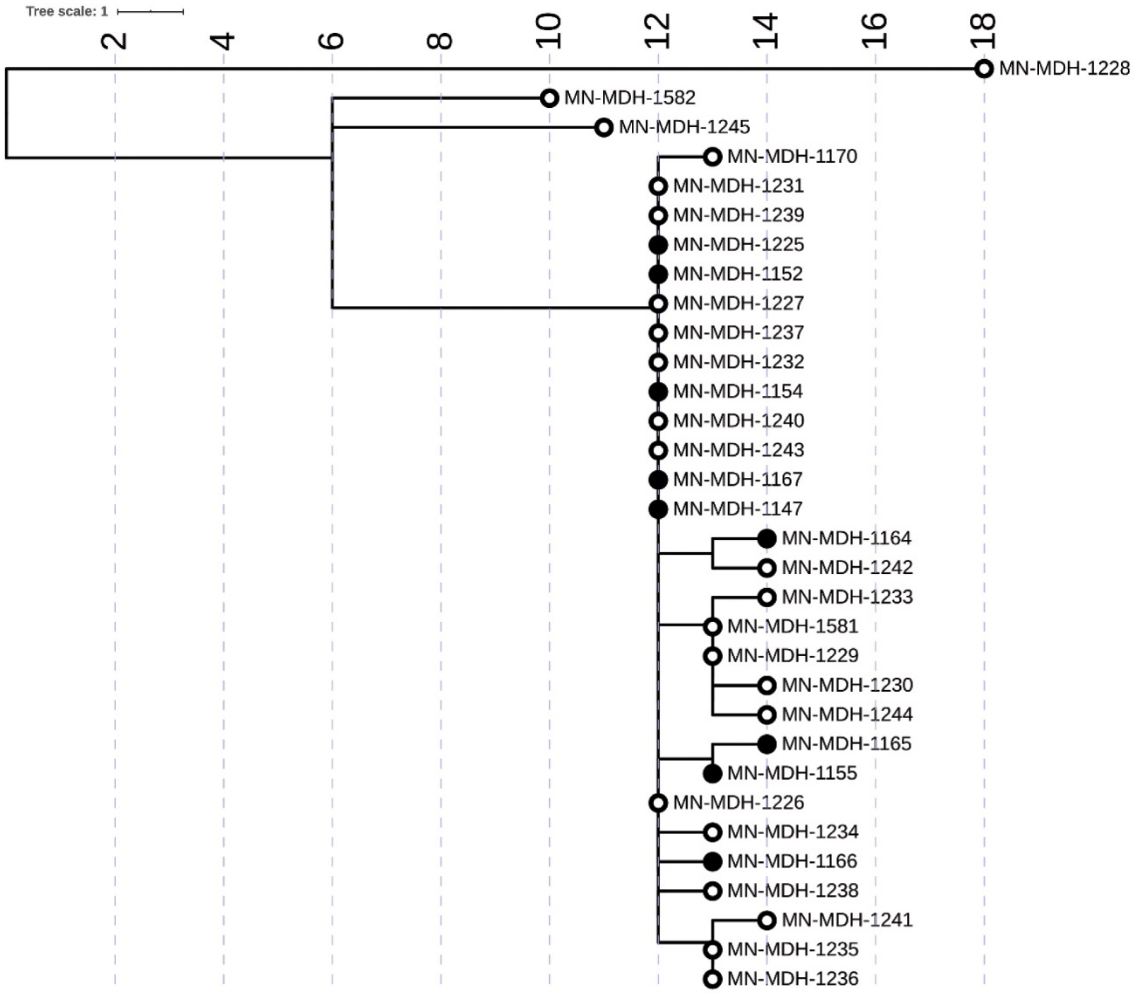

Appendix Figure 2. Phylogenetic tree of SARS-CoV-2 genome sequences associated with LTCF C from April 24 to June 30, 2020. Filled circles represent sequences taken from residents, open circles represent sequences from healthcare workers. IQ-TREE was used with the general time reversible substitution model for tree generation. Branch lengths were scaled to represent number of single nucleotide mutations as shown in the scale key.

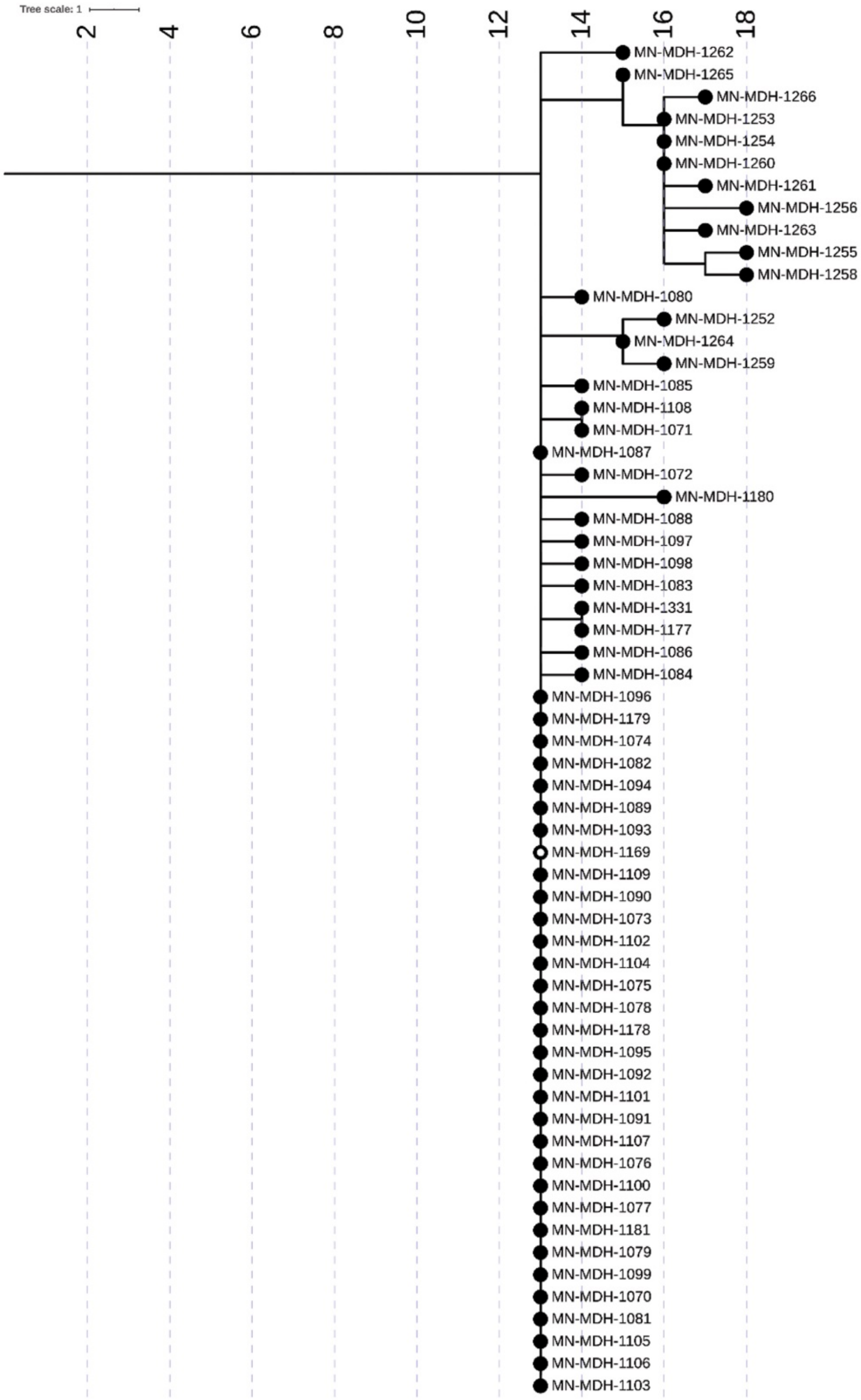

Appendix Figure 3. Phylogenetic tree of SARS-CoV-2 genome sequences associated with Correctional Facility B from May 13 to June 30, 2020. Filled circles represent sequences taken from inmates, open circles represent sequences from facility staff. IQ-TREE was used with the general time reversible substitution model for tree generation. Branch lengths were scaled to represent number of single nucleotide mutations as shown in the scale key.

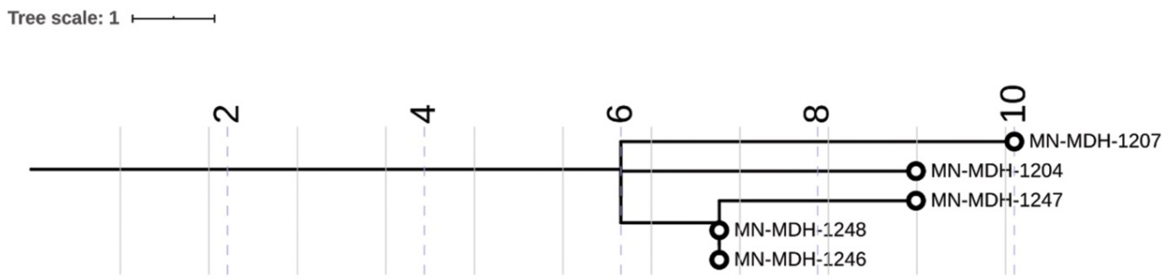

Appendix Figure 4. Phylogenetic tree of SARS-CoV-2 genome sequences associated with Processing Plant B from April 11 to June 30, 2020. Open circles represent sequences from staff at Processing Plant B. IQ-TREE was used with the general time reversible substitution model for tree generation. Branch lengths were scaled to represent number of single nucleotide mutations as shown in the scale key.
